# Intravesical Tension-Free Vaginal Tape Removal: Is There a Single Solution?

**DOI:** 10.5402/2011/343850

**Published:** 2011-04-03

**Authors:** Rhana H. Zakri, Amit K. Patel, Babbin S. John, Nitin C. Shrotri

**Affiliations:** Department of Urology, Kent and Canterbury Hospital, East Kent Hospitals University Foundation NHS Trust, Canterbury, Kent CT1 3NG, UK

## Abstract

Stress urinary incontinence (SUI) affects 10–20% of women in the general population. Surgery for stress incontinence has been performed on women for over a century, but with the advent of new urogynaecological sling procedures for its management, urological surgeons are having to deal with an increasing number of patients presenting with associated complications. With no clarity on the full range of possible complications or certain consensus on their optimal management, the ideal treatment remains a decision for the individual surgeon. In view of this, we felt it of common interest to review the literature for the history of sling procedures, present commonly arising complications, and seek to answer the question in the title.

## 1. Introduction

The anterior vaginal repair was the most popular primary procedure for stress incontinence till the 1970s, but over the last 20 years the operation has been criticised for high recurrence rates [1]. The pubo-vaginal sling procedure was first described by Von Giordano in 1907 using the gracilis muscle to encircle the urethra [2]. By 1942, Aldridge used rectus sheath strips and reported the technique in one woman, describing it as a salvage-type operation in women failing other treatments. As advised by guideline 35 of the Royal College of Obstetricians and Gynaecologists, [1] numerous other materials are now available for use in suburethral slings with autologous material associated with a greater continence rate and fewer complications than either cadaveric or synthetic materials. The authors state that, in general, with all of these materials, the overall risk of vaginal erosion ranges from 0 to 16%, urethral erosion from 0 to 5%, de novo detrusor overactivity from 3.7 to 66% and procedures requiring sling revision or removal from 1.8 to 35%. 10.8% have some voiding disorder post-op, and long-term self-catheterisation is reported in 2% of females. When compared to colposuspension procedures, the suburethral sling carries similar success rates of 85–90% at one year. The college also highlights how the Second International Consultation on Incontinence concluded that suburethral slings represented “an effective procedure for genuine stress incontinence in the presence of previous failed surgery.”

The Prolene (Ethicon) tension-free vaginal tape (TVT), a relatively new anti-incontinence surgical technique, was first described by Ulmsten in 1996. It has since become one of the most frequently performed operations for SUI and is the recommended surgical management according to NICE (NICE clinical guideline 40, 2006). It achieves cure rates greater than 85% [3]. Despite being more expensive than colposuspension, the reduction in hospital stay makes the procedure cost-effective [1]. New minimally invasive techniques are, however, accompanied by a new set of complications including intraoperative perforation of the bladder with the trocar occurring in 5.8% [4, 5]. Lack of familiarity with the cystoscope, not using 70° optics and insufficient distension of the bladder that might hide an injury due to folds of mucosa can cause an occult bladder perforation to be missed [6]. Devices that utilize mesh/slings are also at risk of mesh/tape misplacement, migration and erosion into the bladder, urethra, or vagina [7]. This has been reported in up to 6% of cases [8]. The contact of an intravesical foreign body with urine can often lead to encrustation and usually goes with recurrent or persistent urinary tract infection (UTI) [6]. Mahdy et al. [9] present the third case in the literature of a urethral diverticulum after a TVT procedure. Other complications include peritoneal perforation, acute bowel injury, and denovo presentation for small bowel obstruction where laparotomy revealed a TVT tape violating the peritoneum causing the distal ileum to adhere to the pelvic sidewall [10]. 

Optimal techniques for postcomplication mesh and suture removal have not yet been determined [11] and therefore still remain an operative challenge [12]. The literature regarding the management of iatrogenic foreign body in the bladder or urethra, and especially mesh erosion, is sparse [7]. We further searched the literature for newly published techniques and suggest ways of tackling this problem.

## 2. Discussion

As mentioned, to date there is no consensus about the best approach for treating intravesical tape erosion [6]. Management options of iatrogenic foreign bodies in the lower urinary tract include endoscopic or open techniques [7]. The literature on the intravesical use of holmium laser excision of mesh/suture is again very limited. The holmium laser has established an important role in urology [13] with renewed interest in the use of lasers for minimally invasive treatment of urological disease [14]. The Holmium-YAG laser is a solid-state, pulsed laser that emits light at 2100 nm. This wavelength is strongly absorbed by water and thus laser energy is contained superficially to provide excellent tissue cutting and ablation properties. This provides not only sharp incisions but also simultaneous haemostasis [13, 15]. 

In general two main uses of laser in this context have been described. Giri et al. [4] describe three case histories in which patients previously underwent either a TVT, Burch colposuspension, or Stamey vesicopexy. Patients presented with a range of symptoms such as haematuria, recurrent urinary tract infection (UTI), frequency, urgeny, and incontinence. Upon investigation they were subsequently all found to have eroded nonabsorbable intravesical material. In their series a 365 um holmium laser fiber was inserted through the working channel of a flexible cystoscope and sutures/tape successfully excised using a laser output of 1.0 J per pulse at a rate of 10 Hz. Mean operative time was 15 minutes. Giri et al. concluded that holmium laser excision is a minimally invasive solution to the problem of undetected accidental perforation or erosion following anti-incontinence procedures.

Hodroff et al. [16] describe three females presenting 1-, 2-, and 6 months post SPARC (American Medical systems, In., Minnetonka, Minn, USA) slingplasty with similarly varied complaints. Cystoscopy (flexible cystoscopy and a variety of lens angles with rigid cystoscope) and holmium laser ablation of the intravesical sling was performed. Laser settings of 0.5–8 J at 5–20 Hz were used to divide the mesh at the entrance and exit points. In all cases ablation was performed slightly deeper than the mucosa to allow the mucosa to grow over the puncture site. The authors state the obvious advantage of this technique being less invasive as well as preserving the potential for continued continence. Shrotri et al. [12] describe a 48-year-old female presenting four years after a TVT operation on which cystoscopy revealed encrustation and stone formation over a TVT entering the bladder at 4 o'clock, exiting at the 1 o'clock position. A 365 um laser fibre for Holmium-YAG laser through an operating cystoscope was used to fragment the encrustation and cut the exposed tape flush with the bladder wall. This was then grasped with stent removal forceps and removed transurethrally. The authors conclude that endoscopic holmium laser for fragmentation of encrustation and excision of tape seems to be a safe, effective, and minimally invasive treatment. To our knowledge these are the only case series available in the literature using holmium laser as a method of excision after TVT complications.

Volkmer et al. state that the open suprapubic approach with cystotomy is recommended for removal of TVT tape [17]. Frenkl et al. [7] state, based on the data presented, that their preference for managing mesh within the bladder is also via open cystorrhaphy. To enable complete removal the authors found it easier to “core” the mesh out through the bladder wall or perform a small urethroplasty. In keeping with other reports of early success with endoscopic excision, Frenkl et al. have produced a treatment algorithm for management of iatrogenic foreign bodies in the bladder. They suggest that all patients with a history of recurrent UTIs after surgery for incontinence should undergo careful cystoscopy with a 70° angle lens for the bladder and 0°–30° for the urethra. Those with a suture or <2 staples/screws identified in accessible positions should have an attempt at endoscopic removal. Should this fail or there are either multiple screws/staples or a mesh, then open cystorrhaphy is suggested as the better option.

Use of a suprapubic laparoscopic port in conjunction with transurethral nephroscope has also been described but visibility of mesh located close to the bladder neck was suboptimal [18]. Baracat et al. [19] performed the excision in a similar manner. Kielb and Clemens [20] described a technique which uses laparoscopic scissors via a suprapubic trocar and a cystoscope to visualise and grasp the tape. Pikaart et al. [21] also describe a laparoscopic technique. An intraperitoneal approach was used to enter the retropubic space and remove the sling in their case series of 5 patients. Dissection was completed with a Harmonic scalpel blade as well as blunt dissection to identify the mesh sling retropubically. Average operating time was 104 minutes. Rouprêt et al. [22] have, in 2010, completed a single centre series that claims a complete four-port laparoscopic resection of the TVT. They describe a stepwise technique that initially opens the retropubic space to identify the two ends of the tape. A transparietal tract is then dissected alongside the tape until complete extraction is achieved. Rouprêt et al. describe the role of a concomitant vaginal procedure in instances where the tape is not dislodged using laparoscopy alone. From a series of 38 female patients, the surgeons were able to remove the TVT using laparoscopic techniques alone in 37. The operative time for this ranged from 50 to 240 mins, average 110 mins. They conclude that the technique is safe and feasible but obviate the need for other incontinence procedures for these patients.

Chang and sokol [23] propose a novel minimally invasive technique using the suture passer of the Carter-Thomason CloseSure system for suprapubic assistance during cystoscopic removal of TVT from the bladder. This approach also avoids the need for an open incision or a larger accessory port placed through the bladder.

Huwyler et al. [6] report the first series of five patients with intravesical tape erosion completely removed by standard video-assisted transurethral resection (TUR) under regional anaesthesia. A rigid 24 F cystoscope with 30° telescope was used. Any mesh encrustation was mobilised with endoscopic forceps and the entire penetrating portion removed by the resectoscope loop. Standard electric current, 120 W for cutting and 70 W for coagulation, was in place. The tape was resected until no longer visible or until perivesical fat reached. Mesh and calcifications were extracted with grasping forceps through the cystoscope. In view of complete healing of the bladder mucosa apparent at followup, Huwler et al. consider the transurethral approach a reliable and efficient treatment option for mesh removal from the bladder.

## 3. Conclusion

Iatrogenic foreign bodies in the lower urinary tract can be an early or late complication of female pelvic surgery [7]. Urologists and gynaecologists should exercise caution concerning cases with persisting symptoms resulting from lower urinary tract infection following TVT surgery [3]. It is important for urologists who attempt to remove foreign bodies to be familiar with endoscopic and open techniques for removal, as well as with reconstructive urethroplasties [7]. The endoscopic method can be considered a good alternative for the treatment of late diagnosed vesical transfixion due to synthetic tapes or urethral erosion [19]. 

The Holmium-YAG laser being a multipurpose, multispecialty surgical laser has been shown to be safe and effective for use in the urinary tract [15]. The holmium laser light, carried through small flexible quartz fibres, can also be used with a flexible cystoscope [4] rendering it fast, effective and versatile [11]. In light of the vast number of successful techniques employed for the intravesical removal of TVT, the search to identify one single method is probably unjust. The use of newer endoscopic, minimally invasive techniques undoubtedly offers advantages in terms of post-op recovery and hospital stay, but the use of open techniques for challenging access or large mesh encrustation can certainly not be overlooked. Overall it is important to highlight the diversity of presenting symptoms and therefore have a low threshold for diagnostic cystoscopy in women who have undergone pelvic surgery [7].
